# An Offer to Clergymen

**Published:** 1854-03

**Authors:** 


					﻿AN OFFER TO CLERGYMEN.
This Journal originated in a desire to promote the interests,
the happiness, and well-being of clergymen, and they are in turn
hereby called upon to do something to extend its circulation, if
they approve of its professed object. The editor never so
thoroughly works with a will as when he knows himself paid for
it; he does not profess to work for any one without pay. A free
liberal compensation is a great quickener of both physical and
mental capabilities; it is human nature, hence he has always
insisted on, and paid, the highest charges for medical attention
to the members of his family when absent, or otherwise; know-
ing it to be the most efficient method of securing prompt, willing,
and undivided attention in subsequent emergencies. He knows
that it is customary among regularly educated practitioners of
the old school, not to charge one another or clergymen. He
does not profess this to be his practice. He is satisfied from a
long and wide observation, that it is better both for practitioner
and patient, that there should be a quid pro quo ; the patient
thus retains his independence, which should always be held as
sacred as the birth-right used to be, and to sell it for a mess of
pottage or—physic, is as unmanly, especially when the pottage
never fails to afford comfort and present relief, while the physic
sometimes does.
Therefore, the editor makes the offer, that any who will send
four dollars, will have five copies mailed to any desired address;
or,—
Any one sending three dollars will have three copies sent with
a copy, post-paid, of “ Bronchitis and Kindred Diseases,” eighth
edition, 12mo., 376 pages, 1854. (See Table of Contents on
Cover.) Or,—
Any person sending twenty dollars, will have thirty copies
sent to any desired address.
The editor thinks, that whatever is done to promote the cir-
culation of this Journal will be a public good, for the time has
come when the study of the health must become an essential
branch of primary education ; every year increases the neces-
sity. Our fathers and mothers are still hale and hearty, at
sixty, seventy, or eighty years of age, and yet they never
bothered themselves about the liver and stomach, and digestion,
and brown bread and baths, and hairbrushes; they lived
in blissful ignorance of the locality of the liver, “lights,” or
anything else than the stomach; the whereabouts of “that
animal,” they are regularly and pleasurably reminded of,
three times a day; but not so with us, their degenerate sons,
whose houses are encumbered with double sashes to keep all the
pure air out, while every pains is taken to keep the foul air in ;
with patent shower-baths to chill us to death ; with-hot air fur-
naces to stew us with their stifling, humid heat; with carpets
to hide dust and dirt, to harbor dampness and noxious gases;
and lazy loafing rocking-chairs, to insure three crooks in every
spine; and cushioned ottomans, sofas, lounges, fauteuilles, vis-
a-vis, and a great many other French things, to engender
constipations, piles, fistulas, and lingering death. They indeed
lived in log houses, and sat by roaring wood fires, feasted on
plain “ hog and homminy,” used burnt bread-crust for coffee,
drank “yerb teas” instead of “store tea,” wove flax and
linsey-woolsey, and manufactured what they wore, in the loom-
house, which was considered as indispensable an appendage to
a dwelling, as a kitchen; they went to bed a little after “ candle
light,” and rose at the “ crack of day,” working steady in the
open air from “ morning to night,” beginning their “ Sabbath”
at Saturday sundown, walking across the fields “ to meeting”
on Sunday morning, taking a light lunch at noon, under the*
shady trees, in the church-yard, not so engorging themselves with
a rich Sunday dinner of extra invitingness, as to render them so
insufferably stupid and sleepy during the whole of the afternoon,
as to cause it to be made “ fashionable” to have no “ preaching”
in the afternoon, but to snore it out at home, in blissful security
of its ever being known by the interruption of comers-in, be-
cause it isn’t pious “ to visit on Sundays.”
Persons who have lived thus, regularly, industriously, tem-
perately, religiously, can well afford to be ignorant of the laws
of health, and the precautions necessary to preserve it to mo-
dern livers, for to those who live naturally, nature is a self-
regulator, her instincts are a guide and a safeguard as to
health and disease. But men, whose whole lives are artificial,
must study how to preserve the health under such artificial
circumstances, or the race will die out, and nothing else can
prevent it except intermarriage among hardier tribes—but where
are they to come from ?—civilization is deteriorating the phy-
sique of the nations across the waters, as well as here. Be
assured, reader, that the only remedy for the physical salvation
of man is to secure a practical intelligence as to the laws of his
being, and such is the designed tendency of this Journal.
CARPETS
Are scarcely known in the West Indies, and not one room in a
hundred in Paris has a carpet.
The floors of Parisian dwellings are made of brick, usually
square, and are cleaned with brickdust and water, and are then
rubbed until a mirror-like polish is obtained. See the lower hall
of the Prescott House in Broadway.
SMALL POX—VACCINATION—VARIOLOID.
For the week ending Feb. 11, 1854, in New York city, fifty-
seven persons died of Small Pox, making two hundred and forty-
five deaths, or nearly six deaths a day from that terrible disease
since the 1st of January last. Vaccination is the only protec-
tion, except that of having it in the natural way. Vaccination
is performed at all the Dispensaries, free of charge. I earnestly
advise the permanent residents of New York to apply to some
well known, long established, and reputable physician, whose
duty it is to have always on hand fresh vaccine virus, taken by
them from patients known to themselves personally to be young,
healthful, and of sound constitution and blood,—otherwise Vac-
cination cannot be relied on as a preventive against Small Pox.
What is Vaccination ? What is Varioloid ? How often
ought Vaccination to be repeated ? How may I know for my-
self whether Vaccination has indisputably taken ? A satisfac-
tory answer may be had by purchasing the February number of
Dixon’s “Scalpel,” for 25 cents, from Dewitt and Davenport,
Tribune Buildings, New York ; Peterson, Philadelphia ; Reading
&, Co., Boston. There are two other admirable editorial articles
in this same number, which are of vital interest to the whole
community—What is a Catarrh or common Cold ? What is
Dyspepsia ?
WHEN DOES VACCINATION TAKE ?
On the seventh or eighth day after the “ operation,” we see a
brown centre, of an oval shape, surrounded by pearl colored
dots ; outside of these is a reddish appearance, fading away into
the natural color of the skin ; after the vesicle has passed away,
which is in seven or eight days more, a perfect vaccine crust or
scab presents itself in the place of the vesicle, so that it cannot
be certainly told whether a vaccination has “ taken” until the
fourteenth or fifteenth day. When the pearly dots do not appear
then it has not taken, and the person is either unprotected, or a
previous vaccination has not run out. If after three trials no
pearly dots appear around any sore which may present itself,
then we may feel assured that the person remains fully protected.
				

